# Combined left thoracoscopic and median sternotomy approach to resect aortopulmonary mediastinal paraganglioma following feeding artery embolization: a case report

**DOI:** 10.1186/s40792-022-01534-2

**Published:** 2022-09-23

**Authors:** Kota Itagaki, Hirotsugu Notsuda, Tomoyuki Suzuki, Ryota Tanaka, Hiroki Kamada, Kei Omata, Yuta Tezuka, Hideki Ota, Yoshinori Okada, Yoshikatsu Saiki

**Affiliations:** 1 Division of Cardiovascular Surgery, Tohoku University Hospital, 1-1, Seiryo-machi, Aoba-ku, Sendai, Miyagi 980-8574 Japan; 2 Department of Thoracic Surgery, Tohoku University Hospital, Sendai, Japan; 3 Department of Diagnostic Radiology, Tohoku University Hospital, Sendai, Japan; 4 Division of Nephrology, Endocrinology and Vascular Medicine, Tohoku University Hospital, Sendai, Japan

**Keywords:** Paraganglioma, Aortopulmonary mediastinal paraganglioma, Intrathoracic paraganglioma, Aortic body tumor, SDHD, Pheochromocytoma, Thoracoscopy, Embolization

## Abstract

**Background:**

Aortopulmonary mediastinal paragangliomas are rare. Complete resection of the tumor is desirable regardless of tumor size in view of the risk of sudden death induced by adjacent organ compression and poor prognosis after partial resection or untreated observation. Due to the hypervascularity of the tumor, the risk of intraoperative bleeding is significant, and cardiopulmonary bypass is often required for complete resection.

**Case presentation:**

The patient was diagnosed as having bilateral carotid body tumors and supposedly an aortic body tumor at the age of 43 and eventually underwent resections of bilateral carotid body tumors at the age of 52. The pathology of the carotid body tumors was compatible with paraganglioma on both sides. A familial succinate dehydrogenase subunit D mutation was subsequently identified. Five years later, a contrast-enhanced computed tomography scan showed an enlarged tumor of 45 mm in size in the aortopulmonary mediastinum. Based on the previously known genetic mutation, the tumor was thought to be a paraganglioma. After confirming with an endocrinologist that the aortic body tumor was non-functional, radiologists performed preoperative embolization of the feeding vessels. Subsequently, a surgical team consisting of thoracic and cardiovascular surgeons resected the aortic body tumor using a video-assisted small left thoracotomy approach combined with a median sternotomy approach. The procedure was completed without cardiopulmonary bypass or blood transfusion. The patient was discharged home on postoperative day 9 uneventfully.

**Conclusions:**

After conduction of preceding interventional embolization of multiple feeding vessels, we employed a video-assisted thoracoscopic surgical approach to dissect the aspects of the tumor adjacent to the esophagus, descending thoracic aorta, and left pulmonary artery, followed by a median sternotomy approach to dissect the other aspects of the tumor adjacent to the ascending aorta, aortic arch, right pulmonary artery, and trachea. There have been no reports on scheduled preoperative embolization of feeding vessels to an aortopulmonary mediastinal paraganglioma. Multidisciplinary approach was effective for complete resection of this challenging rare mediastinal tumor.

## Background

Aortopulmonary paragangliomas are rare. Complete resection is preferred because of the risk of sudden death due to (metastasis to other organs or) compression of adjacent organs. However, aortopulmonary mediastinal paraganglioma is deemed difficult to remove surgically because it is adjacent to the aorta, pulmonary artery, trachea, and esophagus, and also because it is a hypervascular tumor. Lamy et al. reported that paraganglioma of the anterior and middle mediastinum is an aggressive tumor, and complete surgical resection, using cardiopulmonary bypass, if necessary, is highly recommended [1]. They also reported a poor prognosis after partial resection or untreated observation. Recently, removal of paragangliomas without the use of cardiopulmonary bypass has been attempted with the development of endovascular therapy and endoscopic surgery [2, 3]. In our present case, we were able to excise the tumor without the use of cardiopulmonary bypass or blood transfusion, by using a combined left thoracoscopic and median sternotomy approach following feeding artery embolization.

## Case presentation

**Fig. 1:**
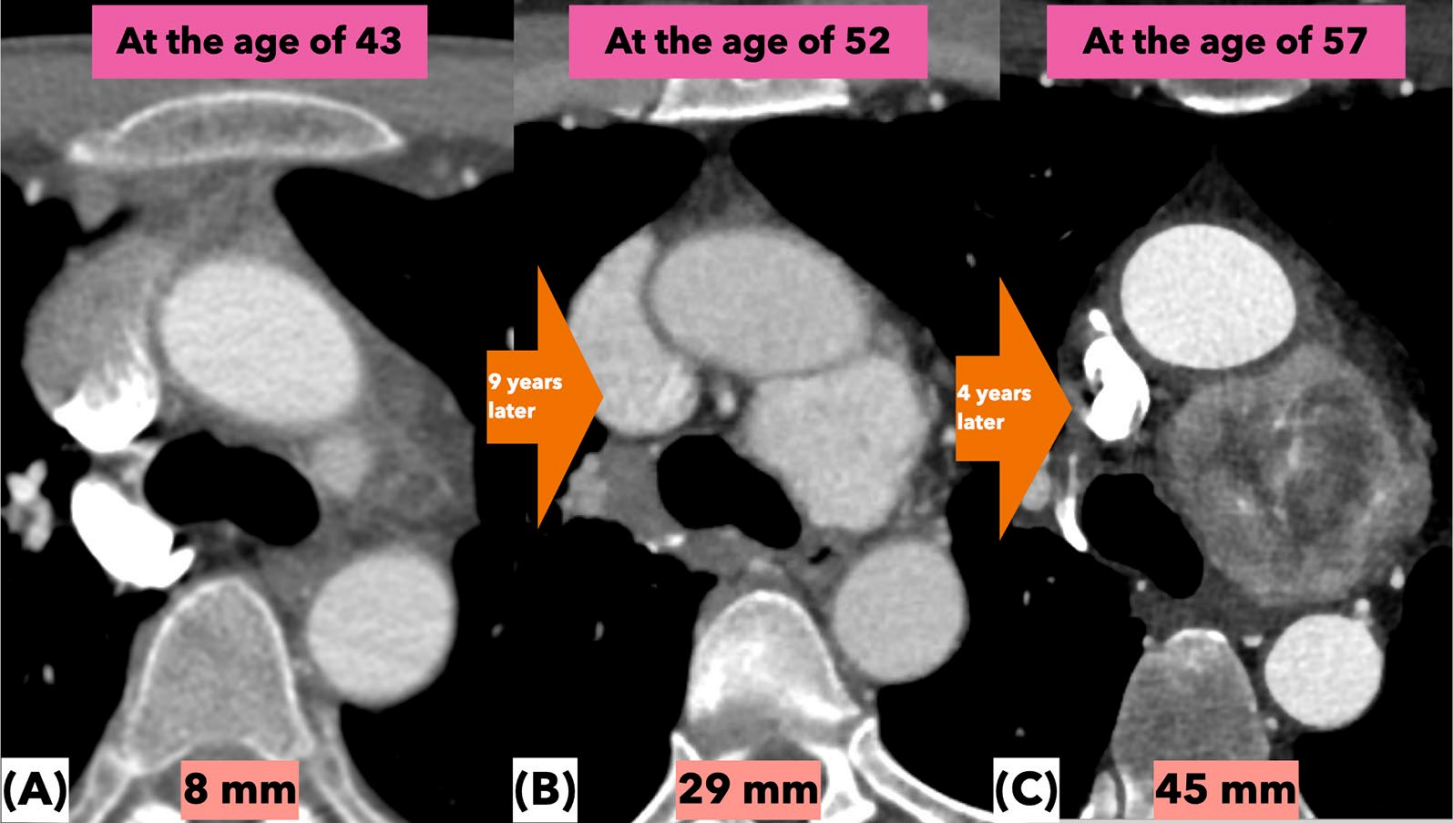
Computed tomographic images depicting serial tumor growth. When the patient was 43 years old, the tumor measured 8 mm in diameter and subsequently exhibited a gradual tendency to expand (**A**). Nine years later, the tumor size increased up to 29 mm (**B**) and it turned out to be 45 mm 4 years later (**C**). As the tumor expanded, the compression of the left bronchus and esophagus progressed over time

**Fig. 2:**
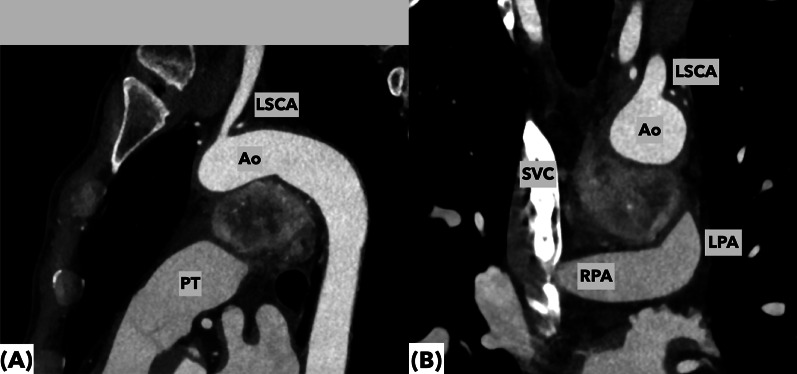
Preoperative computed tomography demonstrating space occupying mass between aorta and pulmonary artery. **A** Sagittal view of the tumor and great vessels was shown. The tumor would be severely adherent to the pulmonary artery trunk and ascending aorta underneath the arch. **B** Coronal view of the tumor and great vessels was shown. The distorted configuration of the aortic arch compressed from underneath and concaved shape of the tumor were well illustrated. These figures raise a great concern about the presence of severe adhesions around the tumor

The patient was diagnosed as having bilateral carotid body tumors and supposedly an aortic body tumor at the age of 43 and eventually underwent resections of bilateral carotid body tumors treated by head and neck surgeons in a local hospital at the age of 52. Following surgery, the patient had slight hoarseness due to residual right recurrent nerve palsy, which was functionally compensated by the left recurrent nerve. The pathology of the tumors was compatible with paraganglioma on both sides and was classified as Shamblin grade IIIa. Subsequently, familial succinate dehydrogenase subunit D (SDHD) mutation was noted. In fact, his family history was positive; his sister had a documented history of skull base tumor surgery and two relatives undergone neck tumor surgeries. Five years later, a contrast-enhanced computed tomography (CT) scan showed an enlarged tumor of 45 mm in size located at the aortopulmonary mediastinum. Sequential follow-up with diagnostic CT scans were retrospectively evaluated (Fig. 1). At the age of 43 years, a hypervascularized tumor of approximately 8 mm was found and inferred to be an aortic body tumor (Fig. 1A), and he, thereafter, was followed by a thoracic surgeon at the same hospital for his convenience. His mediastinal tumor became 29 mm in diameter at the age of 52 (Fig. 1B). Reportedly, the tumor was considered high risk for surgical resection due to its hypervascularity and proximity to vital organs, and it was further followed up at that time. It continued to grow and expanded to 45 mm in maximum diameter by the age of 57. Although there had been no clinical evidence of respiratory distress or dysphagia, progressive compression of the left bronchus and esophagus was revealed by the CT scans. Thus, he was referred to our cardiovascular surgical outpatient clinic for possible surgical management with or without cardiopulmonary bypass support. Based on the previously known genetic mutation, the tumor was thought to be a paraganglioma. We decided to perform surgery because we were concerned about the increased difficulty in removal of the tumor due to prominent vascularity and seemingly tight adhesion to the surrounding organs (Figs. 1C, 2A and B), although he was not being subjected to an imminent risk of sudden death due to a rapid expansion of the tumor as documented in the literature [1].

We proceeded with the functional evaluation of his tumor. ^123^Iodine-MIBG scintigraphy showed focal high tracer uptake in the aortic arch. The tumor was assessed as a non-functional tumor, without catecholamine secretory capacity. A 24-h urine examination revealed that adrenaline and noradrenaline or their metabolites, including metanephrine and normetanephrine, levels were within normal limits. It was inferred that there would be only a negligible risk for hypertensive crisis immediately after selective angiography of the feeding vessels even after surgical manipulation. Thus, preoperative embolization of the feeding arteries was planned as follows.

**Fig. 3:**
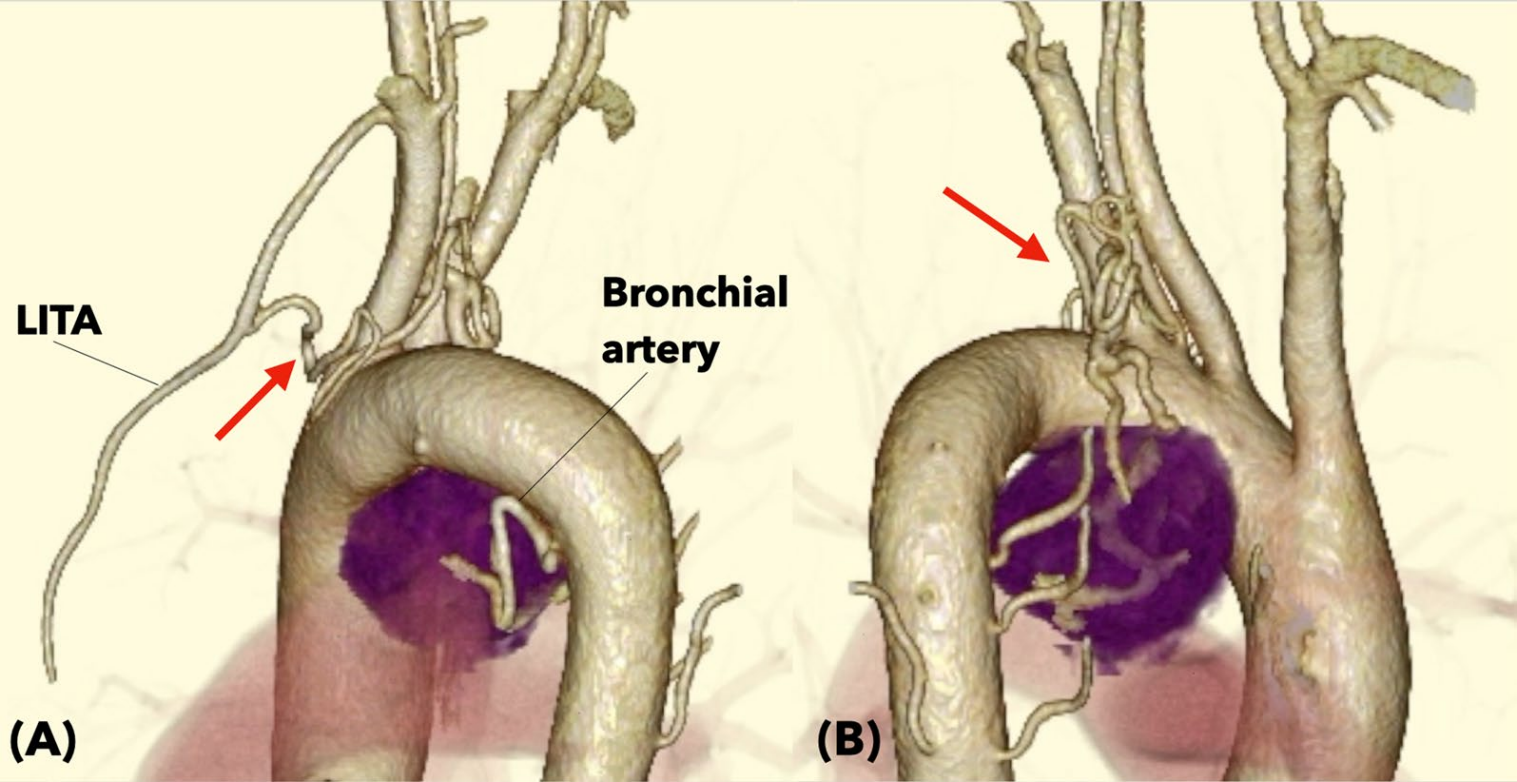
The tumor localization and the routes of major feeding arteries for the tumor are visualized by 3-dimensional CT. The tumor was located in the area surrounded by the aortic arch and pulmonary artery, and was fed by a well-developed mediastinal branch (arrow) of the left internal thoracic artery (LITA) that flew into the right dorsal side of the tumor. The other main feeding artery was derived from the bronchial artery (**B**), which flew from the left dorsal side of the tumor. **A** Viewed from the left lateral side, **B** Viewed from the right lateral side

**Fig. 4:**
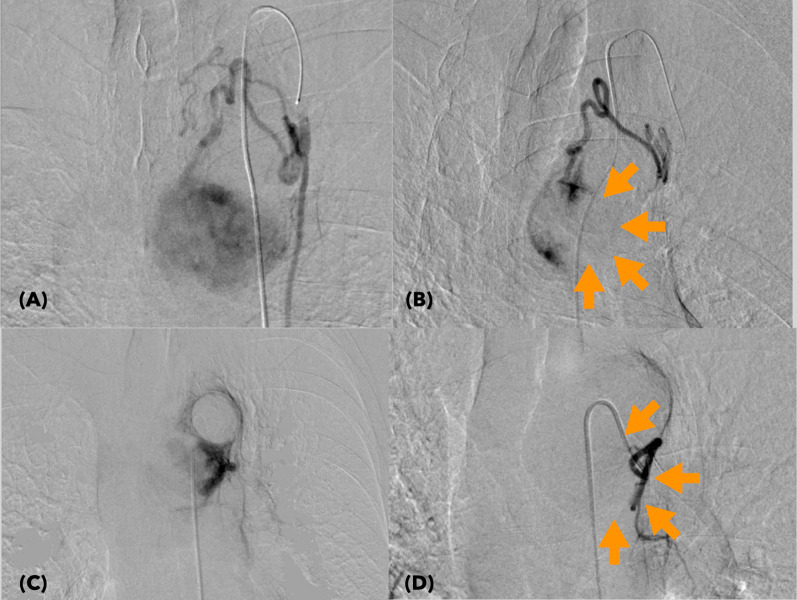
Intraoperative digital angiograms showing embolization of the major feeding arteries. **A** The feeding arteries from the LITA before embolization. The tumor was densely stained. **B** The opacified area decreased in tumor staining (surrounded by arrows) after embolization of the feeding arteries through the LITA. **C** Tumor stain via the feeding arteries from a bronchial artery (BA) was also confirmed before embolization. **D** Following embolization, the tumor staining through BA was completely eliminated. *LITA* left internal thoracic artery, *BA* bronchial artery

CT angiography demonstrated feeding arteries originating from the left internal thoracic artery, bronchial artery, and coronary artery circumflex branches (Fig. 3A, B). The left internal thoracic and bronchial arteries feeding the major component of the tumor were selectively embolized with the fragments of sponzel (Fig. 4A, C). The left circumflex artery was not intervened to avoid potential myocardial infarction following the embolization procedure. Systemic blood pressure of the patient did not change during the procedure. Post-embolization angiography demonstrated no significant opacification of the tumor (Fig. 4B, D).

**Fig. 5:**
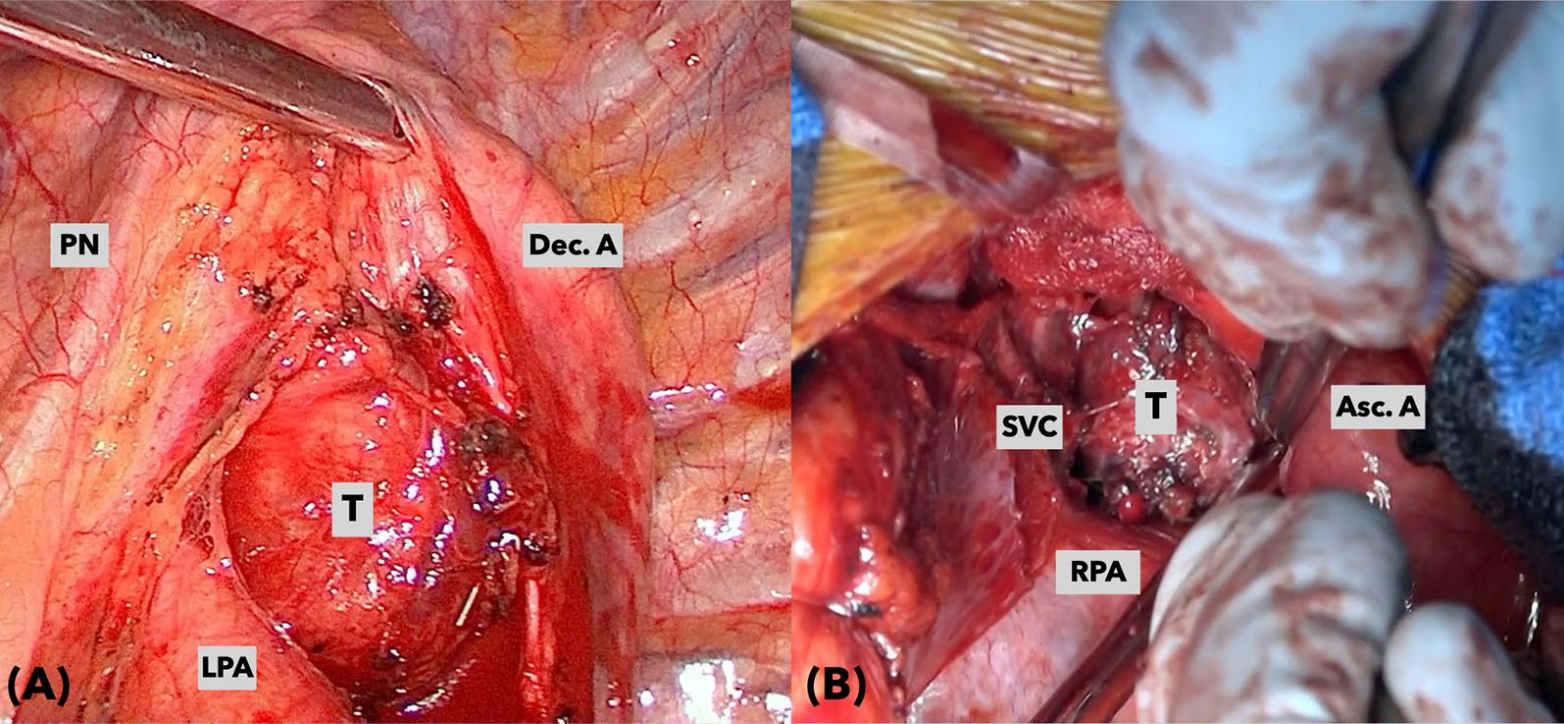
Intraoperative photos. **A** Thoracoscopic image from the left side of the tumor. **B** Direct vision of the right lateral side of the tumor through a median sternotomy. *Asc. A* ascending aorta, *Dec. A* descending aorta, *LPA* left pulmonary artery, *PN* phrenic nerve, *RPA* right pulmonary artery, *SVC* superior vena cava, *T* tumor

**Fig. 6:**
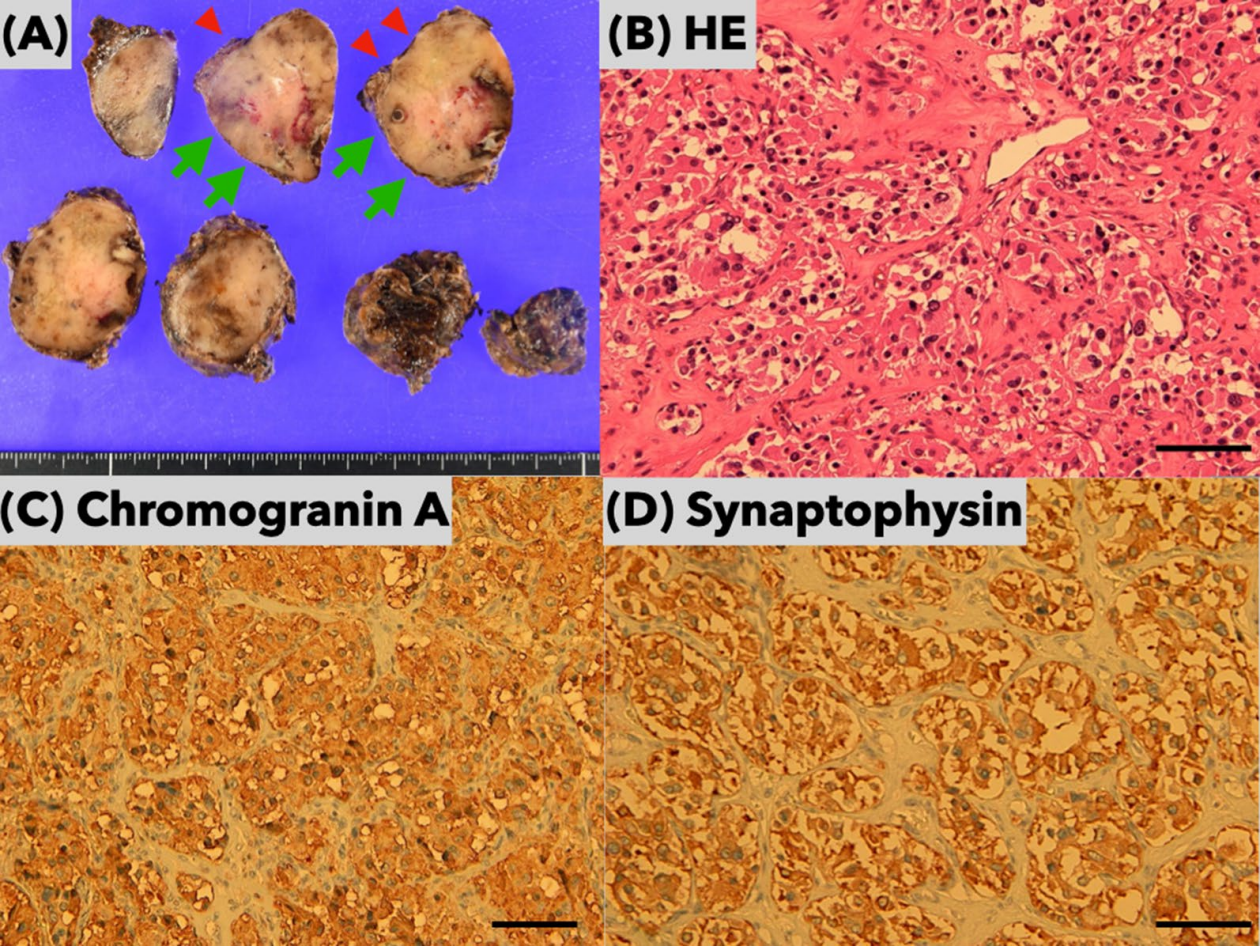
Macroscopic and microscopic findings of the resected tumor. **A** Scale bar is cm. Cross-sectional appearances of the tumor after formalin fixation. Tumor necrosis induced by preoperative feeder embolization was noted as darkish area. Arrowheads point the flattened surface adjacent to the undersurface of the aortic arch. Arrows point the protruding surface of caudal end of the tumor adjacent to concaved pulmonary artery trunk. **B** Scale bar is 100 µm. Nests of cells with abundant cytoplasm were demonstrated (hematoxylin and eosin stain, × 200). **C**, **D** Scale bar is 100 µm. The tumor cells demonstrated positive staining for chromogranin A and synaptophysin (× 200)

On the following day, a surgical team consisting of thoracic and cardiovascular surgeons elected to resect the aortic body tumor using a video-assisted small left thoracotomy approach combined with a median sternotomy approach in an attempt to complete en bloc resection without cardiopulmonary bypass. Thoracic surgeons dissected the tumor of the descending thoracic aorta, left pulmonary artery, left bronchus, and esophagus using a thoracoscopic approach by opening the left fourth intercostal space. Through the fifth intercostal space on the left mid-clavicular line, a single port was created to develop an ideal surgical field. A thoracoscope was inserted via the small thoracotomy site. The tumor was severely adherent to the pulmonary artery trunk and ascending aorta underneath the arch, particularly around the ductus arteriosus. Nonetheless, a preservation of the left recurrent nerve was feasible with thoracoscopic magnified view. The boundary between the adventitia of the great vessels and the tumor was indefinite. Median sternotomy was then performed by cardiovascular surgeons. The root of the feeding vessel derived from the left internal thoracic artery was clipped and the tumor on the right side was dissected. The border between the pericardial reflection and the tumor was easy to observe. LigaSure™ vessel sealing system (Covidien, Dublin, Ireland) was used as an appropriate since there were tiny drainage vessels. Dorsal dissection of the aortic arch was performed; thereafter, the tumor became more mobile. As with the thoracoscopic findings, adhesions around the ascending aorta and pulmonary artery trunk were the most severe, but it was possible to proceed with dissection while preserving the adventitia of the great vessels. (Fig. 5A, B) Intraoperative hemodynamics was stable and bleeding was minimal; therefore, the resection was feasible without the use of cardiopulmonary bypass. Blood transfusion was not required. The operative time was 351 min. The patient was weaned off the ventilator on the day of surgery. He did not develop dysphagia, and the degree of hoarseness unchanged from preoperative status. The patient was discharged on the ninth postoperative day uneventfully. Macroscopic appearance is shown in Fig. 6. The tumor tissue was immunohistochemically stained for chromogranin A and synaptophysin. Both immunohistochemical studies showed positive staining for the neuroendocrinological proteins. Accordingly, definitive diagnosis for paraganglioma was made. The grading system for adrenal pheochromocytoma and paraganglioma, GAPP score, was 2 with Ki-67 of more than 3%. In addition, pheochromocytoma of the adrenal gland scaled score, PASS, was assessed 0 indicating low grade malignancy.

## Discussion

Paraganglioma syndromes 1 through 5 are caused by mutations in the succinate dehydrogenase genes [4]. In terms of extra-adrenal tumors, the frequency is reportedly 10–24% [4]. Rare thoracic paragangliomas have been known to be associated with SDHB, SDHD, and VHL genes. Syndrome 1 is caused by SDHD mutation [5]. SDHD mutations are germline mutations that often occur in patients with multiple primary tumors including head and neck tumors. These tumors have malignant potential. Paragangliomas with SDHD mutations have been reported to have a frequency of metastatic tumors of 1–9% [4].

When the tumor is located between the aortic arch and pulmonary trunk, it is difficult to obtain a complete visualization of the entire tumor in a single view and to safely dissect it. As the tumor is hypervascular, it is difficult to secure a clear operative field if bleeding once occurs, which may affect the maintenance of stable hemodynamics. Some patients require cardiopulmonary bypass during the procedures; therefore, the surgery for an aortic body tumor is often complicated [6]. Wang et al. reviewed 158 cases of thoracic paragangliomas from 132 reports. They reported that intraoperative death due to bleeding was encountered in 3.1% of surgical cases, while in-hospital mortality rate was 6.3% [7]. Carmelina et al. reported that cardiac and pericardial paraganglioma resection may require cardiopulmonary bypass [6]. In general, paragangliomas tightly adhere surrounding organs and have hypervascularity; therefore, the great vessels or the tumor can be damaged during dissection of the adhesion, and the hemorrhage from the vessels or tumor may not be controlled, especially during the resection of aortopulmonary mediastinal paragangliomas. When a degree of damage is severe, and bleeding control is difficult, a patch repair or graft replacement of great vessels along with cardiotomy suction to remove of blood from the surgical field is considered under cardiopulmonary bypass. The use of cardiopulmonary bypass would also reduce the pressure on the great vessels and make it easier and safer to mobilize them and facilitate dissection maneuver around the tumor when efficient dissection is truly difficult due to the adhesion far beyond regular level while maintaining stable hemodynamics during dissecting of the tumor.

Carmelina et al. implicated preoperative tumor embolization to prevent bleeding appears to be an attractive treatment option for some highly vascular tumors, although no practical case undergoing preoperative embolization has been documented for aortopulmonary mediastinal paragangliomas [6]. In fact, there are some reports of cases in which endovascular therapy was documented with the anticipation of bleeding risk reduction before open surgery, although a tumor derived from the aortic body was not included in those literatures [8, 9]. On the other hand, endovascular embolization of paraganglioma has been known to carry a significant risk of hypertensive crisis when a tumor is endocrinologically functional [10]. It is important to assess functionality of a tumor in terms of catecholamine secretion prior to the embolization of the feeding vessels. The patient, therefore, underwent biochemical tests to assure functional negativity. Preoperative embolization of the feeding arteries was then scheduled. In general, measuring plasma fractionated catecholamines for the evaluation of catecholamine secretion for tumors is deemed less sensitive [4]. As a more reliable test, we evaluated catecholamine secretory capacity by measuring urine fractionated metanephrines, normetanephrines, and catecholamines with a 24-h urine test in our present case. An increase in plasma fractioned metanephrines is also reported to have high sensitivity and specificity. It appears to be the first-line clinical test as a denominator for tumor functionality as indicated in the U.S. guideline [10]. This precise diagnostic modality is expected to become available in tertiary referral centers.

Regarding treatment strategy for such tumors, surgical approach needs to be individualized according to the location of the tumor. Recently, with advancements in endoscopic technology, it has been reported that some tumors could be completely resected by thoracoscopic or robot-assisted surgery [2, 3]. One concern is that if there is significant hemorrhage, the field of view may not be secured, and it may be difficult to identify a plane of dissection. Since the tumor was located in an anatomically rare position in our present case, complete thoracoscope or robot-assisted surgery could not be feasible. We elected to employ combined left thoracoscopic and median sternotomy approach taking the degree of expansion of the tumor into account. It was perceivable that there was a blind aspect of the tumor in respective approach as the bulk of the tumor was tightly surrounded by the aorta, pulmonary artery, trachea, and esophagus. In fact, dissection of the tumor on the aorta and pulmonary artery side was not feasible only by thoracoscopic approach due to significant adhesion. Using a 2-way approach, we could safely achieve complete dissection of the tumor free from surrounding vital organs. Since there was no major bleeding or difficulty in developing the surgical field, the use of cardiopulmonary bypass and transfusion were avoided. Nevertheless, we could perform this challenging surgical procedure using a multidisciplinary approach.

## Conclusion

After conducting the preceding interventional embolization of multiple feeding vessels, we used a VATS approach to dissect the tumor adjacent to the esophagus, descending thoracic aorta, and left pulmonary artery, followed by a median sternotomy approach to dissect it adjacent to the ascending aorta, aortic arch, right pulmonary artery and trachea. There have been no reports on scheduled preoperative embolization of feeding vessels for aortopulmonary mediastinal paragangliomas. Multidisciplinary approach was effective for complete resection of this challenging rare mediastinal tumor.

## Data Availability

The data sets supporting the conclusions of this article are included within the article and its additional files.

## Abbreviations

CT: Computed tomography
GAPP: Grading system for adrenal pheochromocytoma and paraganglioma
LITA: Left internal thoracic artery
PASS: Pheochromocytoma of the adrenal gland scaled score
POD: Post-operative day
VATS: Video assisted thoracic surgery
SDHD: Familial succinate dehydrogenase subunit D
